# Different routes of entry of HTLV-1 during infection of primary dendritic cells and CD4+ T cells

**DOI:** 10.1186/1742-4690-11-S1-O77

**Published:** 2014-01-07

**Authors:** Kathryn S Jones, Cari Petrow-Sadowski, Daniel C Bertolette, Nabela Enam, Rachel K Bagni, Stig MR Jensen, David A Davis, Robert Yarchoan, Francis W Ruscetti

**Affiliations:** 1 Basic Research Program, Cancer and Inflammation Program, SAIC-Frederick, Inc., Frederick National Laboratory for Cancer Research, Frederick, Maryland, USA; 2 Cancer and Inflammation Program, Center for Cancer Research, National Cancer Institute, Frederick, Maryland, USA; 3 Protein Expression Laboratory, Advanced Technology Program, SAIC-Frederick, Inc., Frederick National Laboratory for Cancer Research, Frederick, Maryland, USA; 4 Current address: National Institute of Allergy and Infectious Diseases, Bethesda, Maryland, USA; 5 HIV and AIDS Malignancy Branch, Center for Cancer Research, National Cancer Institute, Bethesda, Maryland, USA

## 

Although HTLV-1 is primarily found in T cells in infected individuals, ex vivo cultures of T cells are not readily infected by cell-free HTLV-1. In contrast, cell-free HTLV-1 efficiently infects cultures of primary dendritic cells. Little is known about the productive route of infection of dendritic cells, or the stage at which infection of T cells is blocked. Comparison of primary CD4+ T cells and monocyte-derived dendritic cells (MDDC) exposed to HTLV-1 revealed that, for both cell types, HTLV-1 virions bound to and entered the cells. Entry was dependent on interactions with HSPGs and neuropilin-1, molecules known to be involved in HTLV-1 entry. The observation that cell-free HTLV-1 can enter both types of cells but only efficiently infect DCs suggests that the virus can enter cells by both productive and non-productive pathways. Dendritic cells use a type of constitutive, actin-dependent endocytosis called macropinocytosis to capture antigens, and several viruses use this route to infect host cells. In T cells, macropinocytosis is not constitutive, but can be induced. Studies with inhibitors revealed that HTLV-1 infection of MDDC is markedly decreased when the cells are treated with an actin-depolymerizing agent (cytochalasin D) or a specific inhibitor of macropinocytosis (EIPA). Strikingly, treatment of primary CD4+ T cells with a peptide that induces macropinocytosis dramatically increases infection following exposure to cell-free HTLV-1. These results suggest that HTLV-1 can enter cells by both productive and non-productive pathways, and that altering the route of entry can alter the susceptibility of a given cell type to HTLV-1 infection.

